# DNA topoisomerase I and II expression in drug resistantgerm cell tumours

**DOI:** 10.1038/sj.bjc.6600472

**Published:** 2002-09-09

**Authors:** D M Berney, J Shamash, J Gaffney, S Jordan, R T D Oliver

**Affiliations:** Department of Histopathology and Morbid Anatomy, St Bartholomew's Hospital, Queen Mary's School of Medicine and Dentistry, London EC1 7BE, UK; Department of Medical Oncology, St Bartholomew's Hospital, Queen Mary's School of Medicine and Dentistry, London EC1 7BE, UK

**Keywords:** topoisomerase, germ cell tumour, testis, embryonal carcinoma, seminoma, Ki-67

## Abstract

A small number of testicular germ cell tumours are refractory to current chemotherapy regimens. DNA topoisomerase I is the target for several new drugs and a potential candidate treatment for chemorefractory germ cell tumours. DNA topoisomerase IIα is the target for etoposide, which is currently used regularly in germ cell tumour treatment. The expression of DNA topoisomerase I and IIα were therefore assessed immunohistochemically in a range of testicular tumours, especially those with persistent malignant elements on retroperitoneal lymph node dissection. Pre-chemotherapy orchidectomy specimens were matched with post-chemotherapy retroperitoneal lymph node dissections to examine changes in expression. There was considerable variation in the expression of topoisomerase I in different tumour types. Both yolk sac tumours and teratoma, mature showed universal expression of topoisomerase I, while 38% of seminomas and 30% of embryonal carcinomas were positive. Strong topoisomerase IIα expression was found in embryonal carcinoma. There was a negative correlation between topoisomerase I and IIα expression (*P*=0.004) and downregulation of topoisomerase IIα after chemotherapy (*P*=0.02). Topoisomerase I expression appears to increase in those cases with residual teratoma, mature, but is largely unchanged in those cases remaining as embryonal carcinoma. These results suggest that topoisomerase I inhibitors may be useful in chemorefractory germ cell tumours, especially yolk sac tumours and where there are unresectable residual teratoma, mature deposits.

*British Journal of Cancer* (2002) **21**, 624–629. doi:10.1038/sj.bjc.6600472 www.bjcancer.com

© 2002 Cancer Research UK

## 

The treatment of metastatic non-seminomatous germ cell tumours consists of orchidectomy plus ‘BEP’ combination chemotherapy – bleomycin, etoposide and cisplatin. First line chemotherapy gives a 5-year survival rate for patients with metastatic germ cell tumours (GCT) of over 80% (International Germ Cell Cancer Collaborative Group, 1997). Despite the high success rate of this treatment, the BEP regimen has serious side effects and there is a 1% incidence of drug fatality. Residual masses may remain and it has become common practise to excise these surgically (Einhorn *et al*, 1981). Histology from these masses has a complex range of appearances and the complete resection of embryonal carcinoma (EC) or other malignant elements is important (Fox *et al*, 1993; Fizazi *et al*, 1999; Albers *et al*, 2000). Residual teratoma, mature (TM) is also resected where possible because of the danger of dedifferentiation, or transformation to a variety of other tumour types (Hull *et al*, 1994; Little *et al*, 1994; Michael *et al*, 2000).

Controversy exists regarding the use of further adjuvant chemotherapy (salvage chemotherapy) in these patients, as most are cured by surgery alone (Tait *et al*, 1984; Williams *et al*, 1997). It is clear however that effective salvage chemotherapy does exist for those patients who do relapse (Loehrer *et al*, 1998; Shamash *et al*, 1999). The omission of chemotherapy in these patients would be considered as undertreatment. Patients whose germ cell tumours recur at greater than 2 years from original resection (late recurrences) have a generally poor chemo-responsiveness to current therapies (Michael *et al*, 2000). There remains therefore, a small subgroup of patients who are resistant to current modes of therapy.

The human DNA topoisomerases are members of the gyrase family of enzymes. They are nuclear enzymes, which transiently break and unwind DNA at times when the cell requires access to the genetic material for replication and transcription (Wang, 1996). They are also involved in many cellular activities including chromosome condensation and recombination, DNA repair and segregation during mitosis (Husain *et al*, 1994).

Genes coding for the topoisomerase (topo) enzymes are highly conserved across species. Topo I is targeted by the camptothecins (Takimoto *et al*, 1998) and has been shown to be elevated in many human malignancies (Husain *et al*, 1994; Mcleod *et al*, 1994). Topo II isoenzymes are the targets for the epipodophyllotoxins and for DNA intercalators such as the anthracyclines (Sano and Shuhin, 1995).

Typically, these drugs do not inhibit the free enzyme but stabilise the covalently bound topoisomerase-DNA complex (Rubin, 2000), thus preventing the rejoining of the broken DNA fragments. They act not so much as inhibitors, but as ‘poisons’. The sensitivity of cells to the topoisomerase-targeted drugs is therefore related to the levels of topoisomerase in the nucleus, making immunohistochemical assessment a putative measure of likely cell sensitivity to chemotherapy (Madden and Champoux, 1992). The camptothecins have been investigated recently as possible agents for testicular GCT's (Sano and Shuhin, 1995; Miki *et al*, 1997; Coleman *et al*, 2000).

The aims of this project are to identify the presence of the topo I and IIα in different testicular tumour types, especially those resistant to current therapeutic regimens, to aid the identification of potential targets for salvage chemotherapy using the topoisomerase inhibitors.

## MATERIALS AND METHODS

Cases were identified from the Barts and The London NHS Trust medical oncology database. These included 14 matched cases of orchidectomy plus retroperitoneal lymph node dissection from patients who had residual tumour following their initial course of therapy. Some of the matched pre-chemotherapy orchidectomy tumour blocks were requested from other hospitals, which were largely those participating in the East Anglia germ cell tumour group. Thirteen cases were also identified of retroperitoneal lymph node dissection following an initial course of therapy but where the original orchidectomy was not available. Nearly all these cases had residual malignant or immature elements and therefore represented a selection of the cases which had not responded to conventional therapy. Twelve orchidectomies were also identified from patients who had been diagnosed with seminomas for comparison with the teratoma group. All cases were anonymised.

The cases were all reviewed and the different elements within them identified assisted by immunochemistry where appropriate. Embryonal carcinoma was identified by the immunohistochemical presence of CD30 in the primary tumours, or by histological appearances in the post-chemotherapy cases (Berney *et al*, 2001a). 3 μm formalin fixed, paraffin embedded tissue sections were cut from the selected tissue blocks. Antigen retrieval was used for all of the antibodies used. The antibodies used were topo I (mouse monoclonal antibody Clone ID6, Novacastra), topo IIα (mouse monoclonal antibody Code 3F6, Novacastra), topo IIα (rabbit polyclonal antibody, Novacastra) and Ki-67 (rabbit polyclonal Code AO47, Dako). Ki-67 was included to compare the proliferation index with the topo II enzymes. Normal tonsil was used for a positive control and a negative control was included for each case.

Protocols for immunochemistry were followed according to those used for the topoisomerases in previous studies (Monnin *et al*, 1999). All the antibodies used show nuclear staining. Cytoplasmic staining was disregarded. Protocols for immunohistochemical detection of topo I followed a previous study (Holden *et al*, 1997) and graded the expression of the enzyme in the tissue samples on a subjective scale of 0 to 3+. Staining 2+ or 3+ was counted as positive, and anything less as negative. The expression of the topo II enzyme and Ki-67 was assessed by quantitatively calculating the number of positive cells out of 500 (expressed as a percentage). Fifty cells were counted in 10 random fields within the tumour area. Five hundred cells were counted within each case.

## RESULTS

The expression of topo I in the different tumour types is represented in Table 1

**Table 1:**
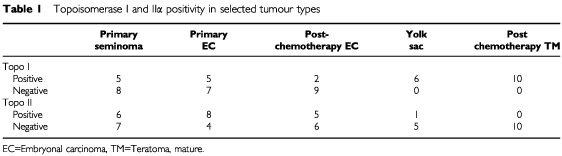
Topoisomerase I and IIα positivity in selected tumour types

**Figure 1:**
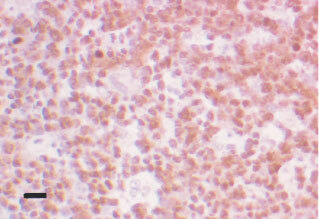
Control showing strong positivity for topo I in a lymphoid follicle. Note the negative macrophages with larger irregular nuclei. Black bar=50 μm.

**Figure 2:**
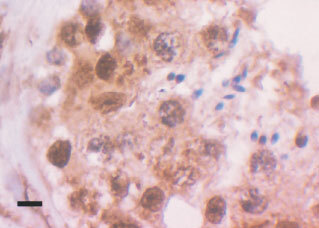
Cross-section of a normal seminiferous tubule showing strongly topo I positive spermatocytes and negative spermatids. Black bar=15 μm.

. Tonsillar tissue, used as a positive control, showed strong staining in lymphoid follicles and in squamous epithelium. Macrophages, which are seen in all normal follicles ingesting apoptotic debris, were noted to be negative (Figure 1

). Background fibrous tissue was also negative for all the tested antibodies. Positive internal controls included lymphocytes especially in the seminomas. Negative internal controls were areas of normal spermatogenesis in the testes where the spermatids, as expected, were seen to be negative (Figure 2

).

**Figure 3:**
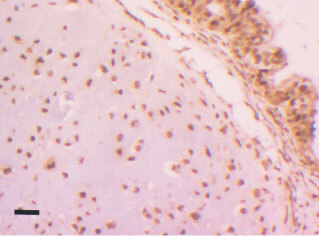
Strong topo I positivity in a mature teratoma after chemotherapy, showing positive chondrocytes and glandular epithelium (top right). Black bar=50 μm.

**Figure 4:**
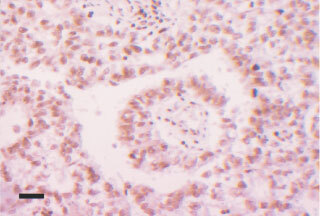
Topo I positivity in a yolk sac tumour. Black bar=50 μm.

Universal strong expression was shown in TM (Figure 3

) and also in yolk sac (Figure 4

). Pre chemotherapy, both EC and seminoma showed similar percentages of cases to be positive (five out of 12 and five out of 13 respectively). After chemotherapy however, only two out of 11 cases of EC showed nuclear positivity. Prominent cytoplasmic staining was noted in the EC group in many cases but this was not counted as positive. Seminomas were chosen as the control group to attempt to determine statistically which of the groups of tumour may respond well to anti-topo I therapy. TM was universally positive for topo I and is significantly different from the seminoma group (*P*<0.003, Fisher's exact test). Yolk sac (YS) was also significantly different from seminoma (*P*<0.018). The changes in expression of topo I after chemotherapy showed that both increases and decreases in expression occurred. Increases occurred in cases which differentiated from EC to TM or YS. Cases with persistent EC either became negative or remained unchanged.

**Figure 5:**
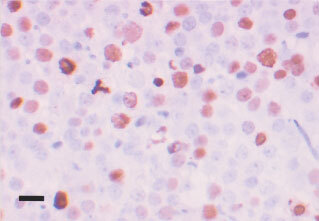
Frequency distribution of topoisomerase IIα index.

Twenty-eight of the total number of specimens were stained with both the polyclonal and monoclonal version of the topo IIα antibody. The tonsil used for control purposes showed very strong nuclear staining in follicles and in basal epithelium. Similar to topo I, spermatids were negative where present in primary orchidectomy cases. The correlation between both antibodies against topo II was strong (*R*=0.908; *P*<0.001) after testing 28 of the cases and therefore the remaining cases were tested with the monoclonal antibody only. The frequency distribution of this antibody was bimodal with 24% used as the cut off between positive and negative cases (Figure 5

**Figure 6:**
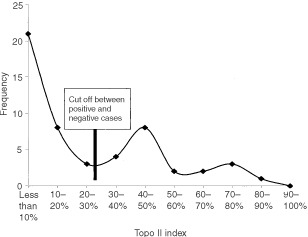
Positive nuclear staining for topo II in a seminoma. Note the strongly positive tripolar mitosis. Black bar=30 μm.

). Topo II positivity is compared with the tumour type in Table 1. The highest topo II index was seen in cases of EC. Lower rates were seen in seminomas (Figure 6

), YS and TM. The TM group showed a significantly lower topo IIα index than seminoma (*P*=0.019, Fisher's exact test). Topo I was also compared with topo II expression (Table 2

**Table 2:**
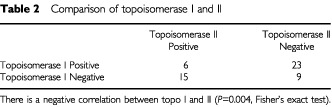
Comparison of topoisomerase I and II

). There was a strong inverse correlation between the two proteins (*P*=0.004, Fisher's exact test), however a few tumours showed dual high expression. Ki-67 showed a positive correlation (*r*=0.69, *P*<0.01) with topo II expression (Figure 7

**Figure 7:**
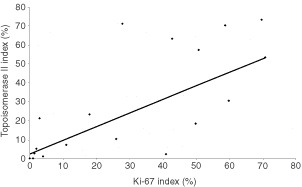
Comparison of the percentage of Ki-67 positive cells with the percentage of topoisomerase IIα positive cells (*r*=0.69, *P*<0.01).

), demonstrating that topo IIα expression is a reasonable measure of proliferating cells. This has been reported in many previous studies (Davidson *et al*, 2000; Gupta *et al*, 2000; Morisaki *et al*, 2000). There was a significant reduction (*P*=0.02, Wilcoxan signed ranks matched pairs test) in topo II positivity after chemotherapy (Figure 8

**Figure 8:**
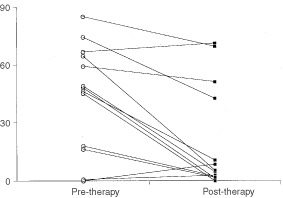
Comparison of topoisomerase II expression in matched samples before and after chemotherapy (*P*=0.02, Wilcoxan signed ranks matched pairs test).

). It was noted that the pre-treatment samples were normally distributed (*P*=0.188, Anderson-Darling test) while the post treatment samples were not normally distributed (*P*<0.001, Anderson-Darling test).

## DISCUSSION

DNA Topoisomerase I has been identified as a molecular target for the plant alkaloid Camptothecin (Husain *et al*, 1994; Holden *et al*, 1997). This is a derivative of *Camptotheca acuminata* (Chinese willow) and shows antitumour activity in human solid tumours including colorectal, prostate and ovarian cancers. These drugs act by preventing the resealing of the DNA, and thus transcription is unable to continue. The greater the amount of topo I a cell has, the more cleavable complexes can be formed within it, and hence, the cell is more drug sensitive (Holden *et al*, 1997). It has also been shown that there is good correlation between the immunohistochemical level of topo I in tissue and catalytic activity (Coleman *et al*, 2000). The increased expression of topo I in tumour tissue may therefore provide a target for selective therapeutic cytotoxicity in other human cancers. This is supported by experimental evidence that Camptothecin-resistant tumour cell lines express reduced levels of topo I (Husain *et al*, 1994).

Topo I has been measured previously in a variety of other malignancies and normal tissues, by immunochemistry, RT–PCR, enzymatic activity and Western blotting. Increased expression has been shown in ovarian epithelial carcinomas (Codegoni *et al*, 1998), transitional cell carcinomas of the bladder (Monnin *et al*, 1999), renal cell carcinomas (Gupta *et al*, 2000) and lung tumours (Mirski *et al*, 2000). Normal topo I levels were seen in the background tissue of the above studies.

Two isoforms of the topo II enzyme exist – alpha and beta. The alpha form is the more important. Its activity is highest during S, G2 and M phases, with low activity during G1 phase. Both the monoclonal and polyclonal antibodies to topo IIα used in this study are specific for the carboxy-terminal of the molecule and do not cross-react with topo IIβ. The activity of topo IIα can be up-regulated by the inhibition of topo I (Whitacre *et al*, 1997). This has an important clinical relevance since, it is conceivable that topo I inhibitors could be used to up-regulate topo IIα, thus making cells more sensitive to topo IIα inhibitors. The results supported an inverse relationship between topo I and topo IIα (Table 2).

Many drugs inhibit topo IIα by stabilising the topo II-DNA cleavable complex (Houlbrook *et al*, 1995). Some topo IIα inhibitors are able to intercalate part of their structure between adjacent DNA bases, e.g. the anthracyclines and anthracene diones which are effectively trapped in the nucleus at a high concentration. They can therefore be administered by a single injection, and will be held in the cell until S phase when topo II activity peaks and they are able exert their effect. Other inhibitors, such as etoposide, do not intercalate and require prolonged administration to increase the chance of exposure to cells during S phase. Seminomas are well known to be sensitive to etoposide (Mencel *et al*, 1994). Evidence suggests that the cellular level of topo IIα determines the degree of drug toxicity (Houlbrook *et al*, 1995). The decreased expression of topo IIα has been shown to be associated with resistance to chemotherapy (Koshiyama *et al*, 2001).

The finding of most interest was the tumour specificity of high topo I expression. Both the TM group, and the yolk sac group show universal strong expression making them potential candidates for treatment with topotecan or irinotecan. The presence of high levels of topo I in TM was unexpected. TM comprises only a small minority of primary GCT's, and these are treated by surgery followed by surveillance. However, after the administration of chemotherapy, the tumours frequently change their morphology and approximately 40% of cases show pure TM. These are routinely resected, though in cases with widespread metastases these foci may be inaccessible to surgery. Despite their quiescent nature, there is a risk of progression and presentation as late recurrences (Michael *et al*, 2000). Thus, if topo I has been upregulated in these foci, administration of an inhibitor may represent an adjunct or alternative to surgical removal in specific cases. Yolk sac tumour is more aggressive than TM but showed similar strong positive expression for topo I. Therefore, in those cases resistant to standard regimens, administration of a topo I inhibitor may be efficacious. Coleman *et al* (2000) investigated the topo I and II expression in seminomas alone. Our results for expression of topo I and II are similar to theirs (6 out of 20 seminomas being positive for topo I in their study and 5 out of 13 in our study). The strong cytoplasmic positivity seen in many cases of EC has been disregarded. However, it has been noted that expression of a cytoplasmic mutant variant of topo IIα has been reported in a lung cancer cell line that was etoposide resistant (Mirski and Cole, 1995). This supports the decision to disregard all cytoplasmic staining.

The primary embryonal carcinomas were the group with the highest expression of topo IIα (8 out of 12) while TM had the lowest (0 out of 10). On comparison with the seminoma group, TM had a significantly lower topo IIα (*P*=0.019). The significant reduction in topo IIα after chemotherapy in matched cases is explained by the transformation to TM from EC. The lack of a normal distribution in the post-chemotherapy cases highlights the variable response to primary chemotherapy.

Ki-67 has been shown to be a useful marker in assesment of likelihood of relapse in metastatic germ cell tumours (Berney *et al*, 2001b). Comparison of Ki-67 with topo IIα shows a good correlation, indicating that topo IIα levels are a fair indicator of proliferating cells. Topo I is thought to be most active in cells with a high S phase fraction as DNA replication forks collide with the stabilised topo I-DNA complex (D'Arpa *et al*, 1990). However, non-replicating cells have been shown to be sensitive to topo I, possibly because of collisions with transcriptional complexes (Morris and Geller, 1996; Wu and Liu, 1997). Therefore in resistant cases, topo I inhibitors may be of great utility.

It should be recognised that upsteam and downstream variables may affect the sensitivity of the tumour to these drugs. The transport proteins Mrp2/Moat (Allen *et al*, 1999) and Brcp/Mxr1 (Koike *et al*, 1997) have been implicated in the efflux of topo I inhibitors and etoposide is a substrate for the cellular efflux protein Mdr1 (Rubin, 2000). However, clinical trials on tumours resistant to conventional chemotherapy and in cases not amenable to surgery are necessary to evaluate the response of these specific types of tumour to the camptothecins.
